# Positive Genetic Correlation Between Yield and Symbiotic Nitrogen Fixation in Pea

**DOI:** 10.1111/eva.70330

**Published:** 2026-10-13

**Authors:** Niall Millar, Clarice Coyne, Stephanie Porter

**Affiliations:** ^1^ Washington State University Vancouver, School of Biological Sciences Vancouver Washington USA; ^2^ USDA Agricultural Research Service Plant Germplasm Introduction and Testing Research Pullman Washington USA

**Keywords:** agriculture, evolution, mutualism, pea, rhizobia, selection

## Abstract

Crop improvement can enhance food security, but side effects, such as trade‐offs between valuable agronomic traits, are common. Likewise, fertilisation helps ensure high yields but can devalue mutualisms with soil microbes that would otherwise be essential for nutrient acquisition. If the need for nutritional mutualisms is reduced in crops, mutualisms could be disrupted by selection relaxation or allocation trade‐offs. Thus, crops could achieve high yields despite or because of, disruption of nutritional mutualisms. Alternatively, the highest‐yielding plants might flourish because they maximise nutrient acquisition from both symbionts and the soil. Here, enhanced mutualism could evolve over the course of agricultural crop improvement. To investigate whether high yields in cultivars and wild accessions are negatively or positively genetically correlated with outcomes in the legume‐rhizobia mutualism, we measured whether yield and symbiosis traits trade off or are positively genetically correlated among cultivars and wild accessions. We measured genetic correlations between yield and mutualism traits in 87 domesticated pea ( *Pisum sativum* ) accessions released before or after 1950 in a common garden agricultural field across 3 years. Seed yield and N_2_ fixation were positively genetically correlated in both pre‐ and post‐1950 accessions. Thus, despite recent selection for pea cultivars with high yields in fertile agricultural conditions, this positive genetic correlation has been maintained. Our findings predict that breeding efforts have the potential to increase both yield and symbiotic nitrogen fixation in pea, enhancing symbiotic mutualism and reducing the proportion of N supplied by fertilisation.

## 1 Introduction

The improvement of crops for human use is a vital and ongoing effort. Over the 20th century, crop improvement has delivered steady yield increases for staple crops like rice (Peng et al. 2000), wheat (Evans et al. 1980) and legumes (Khazaei et al. 2021). There are, however, side effects that arise during crop improvement, such as genetic costs due to inbreeding that can increase the frequency of deleterious mutations (Moyers et al. 2018) and trade‐offs between traits, such as growth and defence against herbivores and pathogens (Córdova‐Campos et al. 2012) (Chen et al. 2015). Further, agricultural conditions could relax selection on traits critical for performance in the wild (Zohary 2004). Genetic costs, trade‐offs and relaxed selection are evolutionary factors that could inadvertently disrupt critical plant mutualisms with symbionts such as arbuscular mycorrhizal (AM) fungi (Jacott et al. 2017; Martín‐Robles et al. 2018) and nitrogen‐fixing rhizobial bacteria (Porter and Sachs 2020) during crop improvement. Alternatively, if these factors do not dominate crop evolutionary dynamics and mutualistic symbiosis remains critical to crop performance and yield in agriculture, crop improvement could enhance mutualism through artificial selection (Porter and Sachs 2020).

The symbiosis between legumes and rhizobia is critical to biological productivity as it contributes a third of human‐consumed protein (Smýkal et al. 2015). In the rhizobial mutualism, legumes host rhizobia in root nodules, wherein these bacteria fix atmospheric nitrogen (N) into compounds usable by the plant in exchange for carbon (C), essentially fertilizing the plant (White et al. 2007). However, N‐enriched conditions in agricultural fields that receive synthetic N‐fertiliser could devalue the N provided by rhizobia (Regus et al. 2016) and relax selection for robust symbiosis (Porter and Sachs 2020). Freely available soil‐N triggers legumes to reduce investment in symbiotic nitrogen fixation and reallocate resources to growth (Nishida and Suzaki 2018). The potential resource allocation trade‐off between N_2_ fixation and growth could result in a scenario where cultivars selected for high yield show low symbiotic function and manifest as a negative genetic correlation between these traits among cultivars. Yet, the potential for genetic correlations between yield and symbiosis traits has not typically been considered directly in crop breeding decisions (but see Jacott et al. 2017).

Understanding of the strength and direction of genetic correlations between yield traits and symbiosis traits is needed to predict how selection for high yield impacts symbiosis. If yield and symbiosis traits trade off, this could generate negative genetic correlations between them. If plant resource allocation to seeds comes at the expense of allocation to N_2_ fixing root nodules, selecting for plants with higher yields could indirectly select for lower symbiotic N_2_ fixation (Tamagno et al. 2018). However, an opposite scenario wherein yield traits and symbiosis traits are positively correlated is also possible. If symbiotic N_2_ fixation instead enhances seed production by boosting total nitrogen acquisition, selecting for plants with higher yields might indirectly select for plants that receive a greater proportion of their N from rhizobia. A third possible scenario is that yield and symbiosis traits show no genetic correlation such that breeding to enhance yield does not affect the evolution of rhizobial mutualism (Herridge et al. 2005; Sikinarum et al. 2007). It is possible to produce pea lines that have both increased yields and N_2_ fixation potential, at least when crossing with nodulation mutants (Dhillon et al. 2022). In fact, some high yielding domesticated soybean have higher N_2_ fixation than their wild relatives (Muñoz et al. 2016), though they may not receive as much net growth benefit from rhizobia as wild soybeans (Millar et al. 2023). Understanding which of these hypotheses is best supported will help to inform strategies for enhancement of the rhizobial mutualism in agriculture.

Improvements to the rhizobial mutualism could create more sustainable legume crops that require less N fertilisation for the same or greater yields, yet plant‐microbe mutualisms have unrealised potential as breeding targets (Bennett et al. 2013; Dwivedi et al. 2015; Rog et al. 2026). Crop breeding for N_2_ fixation capacity in soybeans, common beans and pigeon pea all shows promise for increasing yields (Herridge et al. 1998; Nicolás et al. 2002; Alves et al. 2003). Peas have been characterised as poor N fixers in comparison to other legume crops (Liu et al. 2019), so they stand to reap major benefits from similarly targeted breeding programs.

To uncover how evolutionary constraints may shape beneficial symbiosis over the course of domestication and crop improvement, we characterise diverse pea accessions under field conditions to distinguish, (1) whether genetic correlations between yield traits and symbiosis traits are: (a) negative, consistent with resource allocation trade‐offs or relaxed selection on symbiosis, (b) positive, consistent with indirect selection for increased symbiotic function or (c) undetectable, consistent with no impact of N_2_ fixation on yield. To understand how crop improvement affects trait correlations we asked, (2) whether the genetic correlation between yield and symbiosis traits changed over the course of crop improvement during the 20th century. To answer these questions, we used pea accessions from the ‘More Pea, More Protein, More Profit’ (MP3) *Pisum sativum* diversity panel (Jermyn and Slinkard 1977; Cheng et al. 2015). This panel of 487 pea accessions was selected for high seed protein concentration. We focused our investigation of symbiosis traits on a subset of 87 accessions from this population selected to span the diversity present in the MP3 panel, as well as 11 wild pea genotypes.

## 2 Methods

### 2.1 Pea Germplasm, Location and Experiment Design

The Pacific Northwest, USA is a major centre of pea agriculture, making it a useful context in which to investigate key pea trait correlations. As part of a broader study to identify pea germplasm useful for breeding peas with high seed protein concentration, 487 MP3 accessions (Jermyn and Slinkard 1977; Cheng et al. 2015) were planted in a randomised complete block design with three replications, at the USDA Central Ferry Research farm (46°39′5.1″ N, 117°45′45.4″ W; 198 m asl) in the Palouse region of eastern WA in 2019, 2020 and 2021 (Sari et al. 2025). We present data for 87 accessions from this broader study of domesticated pea, *P. sativum*, that we selected to represent the major groups of pea diversity presented in Holdsworth et al. (2017) and Cheng et al. (2015) as well as 11 accessions of domesticated pea's closest wild relative, *Pisum sativum elatius* (Kosterin et al. 2010) (Table S1).

Prior to planting each year, soil samples were taken across the three adjacent spatial blocks in the agricultural field, homogenised and tested for N, P, K, S, B, Zn and Mn content (Soiltest Farm Consultants, Moses Lake, WA). Based on deficiencies detected by the tests, fertilisers were applied to the field each year before planting (Table S2). Additionally, pre‐emergence herbicide (α, α, α‐trifluoro‐2,6‐dinitro‐*N,N*‐dipropyl‐P‐toluidine; Treflan, Dow Chemical), was applied to the soil prior to planting each year. As is standard for pea cultivation in the region, the seeds were pre‐treated with fungicides (mefenoxam [13.3 mL active ingredient (a.i.) 45 kg^−1^], fludioxonil [2.4 mL a.i. 45 kg^−1^] and thiabendazole [82.9 mL a.i. 45 kg^−1^]) insecticide (thiamethoxam [14.3 mL a.i. 45 kg^−1^]) and sodium molybdate (16 g 45 kg^−1^).

The experiment was planted on the 16th, 8th and 1st of April across the 3 years of the experiment, respectively, to optimise weather conditions for planting. In each of the 3 years of the experiment, there were three adjacent blocks containing 487 plots each, one for each accession, for a total of 1461 plots per year. We focused on a subset of 87 accessions of domesticated pea from this larger experiment (Table S1), so each year we sampled from 87 randomly placed plots per block, for a total of 261 plots per year. In 2020, there were 12 additional plots per block for the 11 wild *P. sativum elatius* accessions and a non‐nodulating *P. sativum* accession, Frisson p56 (Sagan et al. 1993), for a total of 297 plots in that year.

Each plot was a 152 by 30 cm space sown with 30 seeds. The seeds were mechanically drilled in two 152 cm lines with 30 cm centre spacing. Plots in the same row were 152 cm apart and adjacent rows were 100 cm apart. Each row contained 30 plots (excluding the final row in each block) and there were 17 rows per block, for a total of 51 rows per year (Figure 1). Each row was irrigated 10 min per day from drip tape buried 15 cm under the centre of each plot. On average, 24.4 plants germinated and survived per plot. Peas have been grown at this site for 31 years, in rotation with winter wheat, spring wheat and Brassica cover crops. The soil contains rhizobia compatible with peas, so no rhizobial inoculation was applied in this experiment.

**FIGURE 1:**
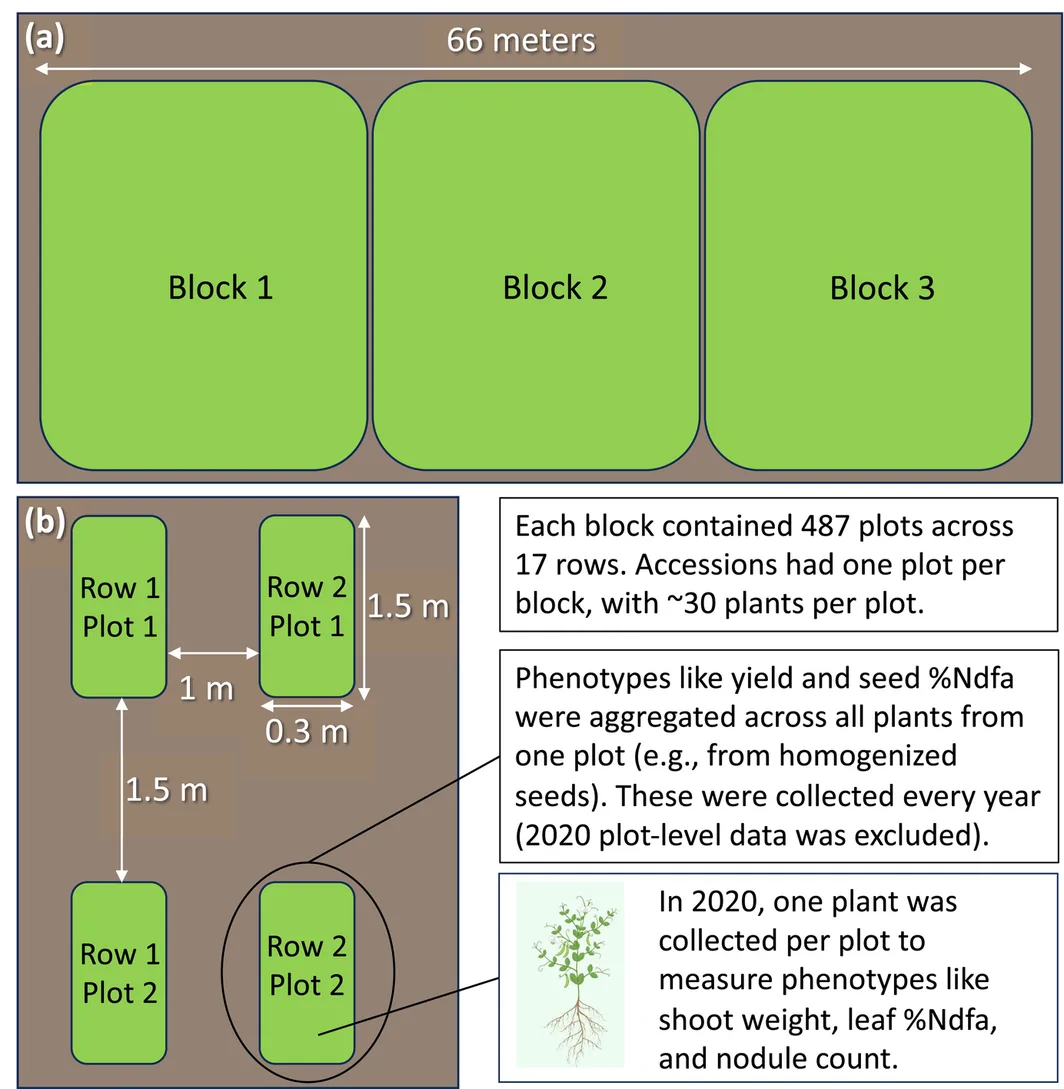
Experiment design at Central Ferry Research Farm. (a) In each of 3 years MP3 pea accessions were grown in a randomised complete block design with three blocks in different locations on the farm each year. (b) Each 30 cm × 150 cm plot was planted with ~30 seeds in two lines. Plot‐level data (seed %Ndfa, yield, N yield, C yield, seed weight %N, seed weight %C, protein yield, seed % protein and seed δ13C) were aggregated across all plants in a plot. Plant‐level data (leaf %Ndfa, shoot weight, shoot weight N, shoot weight fixed N, root weight, nodule count) was collected only in 2020 and plot‐level data from this year was excluded from our main analyses due to storm damage.

### 2.2 Yield Traits

To measure peas' yield traits, we took measurements aggregated across all individuals in an experimental plot for all 3 years of the study (i.e., ‘Plot‐aggregated phenotypes’). Each year the plots containing mature pea seeds were manually harvested in order of maturity (between the second week of July and the second week of August), threshed and the seeds blown clean. Yield was measured as the total weight (g) of threshed seeds from a plot. Seeds from a plot were ground to powder, homogenised and analysed for seed protein concentration (SPC) with near‐infrared (NIR) spectrometry (Matrix I, Bruker Co.).

In addition to these plot‐aggregated phenotypes, we also sampled one individual plant per plot in 2020 (i.e., ‘Individual plant measurements’). To measure yield traits for individual plants, on the 19th and 20th of May, 2020, 6 weeks after planting, we sampled a single plant from each replicate plot for 87 accessions, the 11 wild accessions and the non‐nodulating mutant. Where possible, we chose a plant that was spatially separate from others in a plot, to minimise damage to adjacent plants. We carefully excavated the shoot and root system, washed roots with water and separated them. We wrapped individual root systems in wet paper towels and transported them in chilled coolers before counting the root nodules. We then dried the shoots and roots and weighed them.

In the summers (April through August) of all 3 years, the average monthly rainfall was 1.19 cm. However, in May, 2020, the experiment experienced an extreme weather event when a storm deposited 7.79 cm of rainfall, mostly between the 17th and 20th of the month, giving May 2020 the highest rainfall in the three summers of the study. Storm damage to each plot was assigned a damage severity value (1: No damage—5: severe damage), which was strongly correlated with yield (Pearson correlation coefficient = −0.64, *p* = 0, S1). Due to this damage, we excluded the Plot‐aggregated phenotypes from 2020 from our analysis. The Individual plant measurements were taken from plants harvested at the start of the storm in May of 2020, before the damage occurred, so this data was retained in the analysis.

### 2.3 Symbiosis Traits

Our primary measure of symbiotic nitrogen fixation was percent nitrogen derived from the environment (%Ndfa) in pea tissue, which we calculated using the ^15^N natural abundance method (Unkovich et al. 2008),

$$\%\text{Ndfa}=\frac{\delta^{15}N\;\text{of reference plant}-\delta^{15}N\;\text{of}\;N_{2}\;\text{fixing legume}}{\delta^{15}N\;\text{of reference plant}-B}\times \frac{100}{1}$$

Here, $\delta^{15}N\;\text{of reference plant},$ refers to $\delta^{15}N$ for a pea plant that is entirely dependent on soil N and *B* is the δ^15^N measured for a pea plant that is entirely dependent on symbiotic N_2_ fixation.

We encapsulated 1–1.5 mg of ground, homogenised seed from each plot for stable isotope analysis at the WSU Stable Isotope Core Laboratory in Pullman, WA. Samples were analysed for δ^15^N, % N, δ^13^C and % C, by weight. The samples were converted to N_2_ and CO_2_ with an elemental analyser (ECS 4010, Costech Analytical, Valencia, CA); these two gases are separated with a 3 m GC column and analysed with a continuous flow isotope ratio mass spectrometer (Delta PlusXP, Thermofinnigan, Bremen). Using the % by weight values for protein, N and C, we calculated the absolute weight (g) of each of these properties, based on total seed weight. We used the plot‐aggregated seed δ^13^C values as estimates of the plants' water use efficiency (Kaler et al. 2018).

Initial calculations of %Ndfa using the mean δ^15^N value of the non‐nodulating mutant as the reference value and the peas' lowest δ^15^N value (−2) as the *B* value, returned some %Ndfa values > 100 and < 0. In order to obtain %Ndfa values between 0 and 100, we used the δ^15^N value of barley (8.9) (Unkovich et al. 2008) and a *B* value of −4.5. Therefore, we primarily interpret our %Ndfa values to show relative differences between our samples rather than precise absolute values, since different constants will change the magnitude, but not the ranking of %Ndfa values (Cox et al. 2022). We measured the plot‐aggregated %Ndfa of the ground seeds from each plot for each of the 3 years of the study. In 2020, we also estimated %Ndfa, as above, with a 1–1.5 mg sample of tissue from the apical leaf from the single plant we collected from each plot. We also counted root nodules for this plant.

### 2.4 Genetic Correlations

The complete randomised block design of this experiment allowed us to partition total phenotypic variance (*V*_*p*_) into genetic variance components (*V*_*G*_) and environmental variance components (*V*_*E*_) (Hill and Mackay 2004). We thus estimated the proportion of variation in the peas' yield traits and their symbiosis traits attributable to: (1) *V*_G_ as broad sense heritable genetic variation among accessions and, (2) *V*_E_ as trait plasticity of accessions due to both spatial environmental variation among blocks in the field (e.g., due heterogeneity in soil chemistry; Table S2) and temporal environmental variation among the years of the experiment (e.g., due to weather conditions that year; Figure S1).

To calculate genetic correlations among plot‐aggregated phenotypes, we applied the following generalised linear mixed model (GLMM) to each of our measured traits (Skovbjerg et al. 2020), which we will collectively call ‘Model A’:

$$T_{\mathrm{ijk}}=\mu +A_{i}+R_{j}+Y_{k}+\varepsilon_{\mathrm{ijk}}$$

where *T*_*ijk*_ is a variable representing a given trait, *μ* is the overall mean, *A*_*i*_ is the random effect of the *i*th accession (87 levels), *R*_*j*_ is the random effect of the *j*th experimental block (3 levels per year), *Y*_*k*_ is the fixed effect of experiment year (2 levels) and *ε*_*ijk*_ is the residual error term of the *i*th accession in the *j*th block in the *k*th year. We excluded data from 2020 in these models due to extensive storm damage, keeping data from 2019 and 2021. To estimate the best linear predictors (BLUPs) for each trait, we extracted the random effects estimates for accession from each model (Skovbjerg et al. 2020).

Pea cultivars are self‐fertile inbred lineages, so correlations among accessions' traits can be viewed as ‘broad sense genetic correlations’ sensu Shaw (1986), as additive genetic effects remain confounded with maternal genetic and epistatic variance. Therefore, the genetic effects we estimate represent an upper bound on the additive genetic variance. To measure broad sense genetic correlations, we generated Pearson correlation coefficients between the yield trait BLUPs and the symbiosis trait BLUPs. To account for multiple tests for genetic correlations between yield traits and symbiosis, we subjected all *p*‐values for genetic correlations to a sequential Holm‐Bonferroni correction.

To test yield‐symbiosis correlations over the course of 20th century crop improvement, we separated accessions into those introduced before vs. after 1950. We then generated separate pre‐ and post‐1950 BLUPs, using Model A. As above, we calculated Pearson coefficients for genetic correlations between yield traits and symbiosis traits. We used the Cocor package (version 1.1‐4, Diedenhofen and Musch 2015) to test whether genetic correlations differed between the age groups. Cocor uses Fisher's *z* to test whether there are differences between correlation coefficients and whether the difference between the R‐squared values exceeds Zou's confidence intervals (Zou 2007). Fisher's *z* and Zou's confidence intervals test for differences in correlation strength, that is, the distance of points from the line of best fit. To test for differences in the slopes of the pre‐ and post‐1950 yield‐symbiosis correlations, we used a GLMM with the seed %Ndfa BLUPs as the response variable, the fixed effect of the yield BLUPs, age group, their interaction and the random effect of experimental year. We used a likelihood ratio test (LRT) to determine the significance of the yield BLUPs‐age group interaction, from which we inferred whether the slope of the yield‐symbiosis correlation differed between age groups. To explore how influential a set of particularly low values were, we conducted a post hoc test where we removed the five lowest yielding pre‐1950 accessions and re‐ran the pre/post‐1950 correlation comparisons.

In addition to comparing yield‐symbiosis correlations between the age groups, we tested the effect of age group on individual traits to see if, for example, seed yield means differ between pre‐ and post‐1950 accessions. We used the following GLMM for each trait (Skovbjerg et al. 2020), which we will collectively call ‘Model B’:

$$T_{\text{ijkl}}=\mu +A_{i}+R_{j}+Y_{k}+G_{l}+{\mathrm{YG}}_{\mathrm{kl}}+\varepsilon_{\text{ijkl}}$$

where *T*_*ijk*_ is a variable representing a given trait, *A*_*i*_ is the random effect of the *i*th accession (87 levels), *R*_*j*_ is the random effect of the *j*th experimental block (3 levels per year), *Y*_*k*_ is the fixed effect of the *k*th experiment year (2 levels), *G*_*l*_ is the fixed effect of an accession's age group (pre‐ or post‐1950), Y*G*_*kl*_ is the interaction between age group and experimental year and *ε*_ijk_ is the residual error term of the *i*th accession in the *j*th block in the *k*th year and *μ* is the overall mean. We used LRTs to generate *χ* ^2^ values and determine whether trait means were significantly affected by age group.

Traits that were only measured on individual plants in 2020 were modelled separately. For the 2020 single‐plant data (including shoot weight, root weight, nodule count, leaf %Ndfa, leaf % by weight N and total N content), we used Model A for generating BLUPs and Model B to test the significance of age group on traits and here included three levels within *Y*_*k*_, the fixed effect of experiment year. We removed the experimental year effect for the 2020‐only models.

In some contexts, the use of BLUPs to estimate evolutionary parameters may be anticonservative (Hadfield et al. 2010). To confirm the genetic correlations we generated using BLUPs with a different statistical approach, we used bivariate models (Hadfield et al. 2010), which we will collectively call ‘Model C’:

$$M_{\text{ijkl}}=\mu +A_{i}\delta_{i}+R_{j}+Y_{k}+G_{l}+\varepsilon_{\text{ijkl}}$$

where *M*_*ijkl*_ is a matrix containing seed %Ndfa and a yield trait, *A*_*i*_ *δ*_*i*_ is the random effect of the *i*th accession with an unstructured covariance matrix, *R*_*j*_ is the random effect of the *j*th experimental block (3 levels per year) with a diagonal covariance matrix, *Y*_*k*_ is the fixed effect of the *k*th experiment year (2 levels), *G*_*l*_ is the fixed effect of an accession's age group (pre‐ or post‐1950), *ε*_*ijk*_ is the residual error term of the *i*th accession in the *j*th block in the *k*th year with a diagonal covariance matrix and *μ* is the overall mean. Models were run in sommer (Covarrubias‐Pazaran 2016). We took correlation coefficients between seed %Ndfa and the yield traits from the variance–covariance matrix for the accession random effect. To determine the significance of these correlations, we performed LRTs, using null models where the among‐accession covariance structure (*δ*_*i*_) was limited to 0 with a constraint matrix. Additionally, we used model C to evaluate pre‐ and post‐1950 age group correlations using LRTs with null models with the age group fixed effect removed. We thus used model C to re‐analyse the data from 2019 and 2021. However, the sommer package can fail to properly estimate correlations in some cases, possibly due to low variance or insufficient data, returning correlation coefficients greater or less than 1 (Collet et al. 2023). This occurred for the pre‐1950 subset and for the plant‐level data from 2020, so we do not report correlation coefficients for these groups.

All analyses were conducted in R version 4.2.2 (R Core Team 2014). We used the glmmTMB package (version 1.1.14, Brooks et al. 2017) to run models A and B and the sommer package (version 4.4.6, Covarrubias‐Pazaran 2016) to run model C. We used the DHARMa package (version 0.4.6, Hartig 2022) to check model assumptions.

## 3 Results

### 3.1 Genetic Correlations Between Yield and Symbiosis Traits

In 2019 and 2021 Model A revealed positive genetic correlations between seed %Ndfa and the following plot‐aggregated traits: yield (*r*(85) = 0.56, *p* < 0.001, Table S3, Figure 2a), seed protein weight (*r*(85) = 0.52, *p* < 0.001, Table S3, Figure 2b), seed N weight (*r*(85) = 0.61, *p* < 0.001, Table S3, Figure 2c) and seed C weight (*r*(85) = 0.75, *p* < 0.001, Table S3, Figure 2d). However, seed %Ndfa did not correlate with seed protein concentration, % by weight N or % by weight C, nor with seed δ^13^C. Patterns of significance based on BLUPs were recapitulated in bivariate models from Model C, though correlation coefficients from bivariate models tended to be higher (Table S4). The total weight of N derived from symbiotic fixation (Total fixed N weight), calculated from seed %Ndfa and total seed weight (yield), was positively correlated with yield (*r*(85) = 0.94, *p* = 0, Figure 3a), seed protein weight (*r*(85) = 0.89, *p* = 0, Figure 3b), seed N weight (*r*(85) = 0.99, *p* = 0, Figure 3c) and seed C weight (*r*(85) = 0.85, *p* = 0, Figure 3d). Total fixed N weight was uncorrelated with seed protein concentration, % by weight N, % by weight C or seed δ^13^C.

**FIGURE 2:**
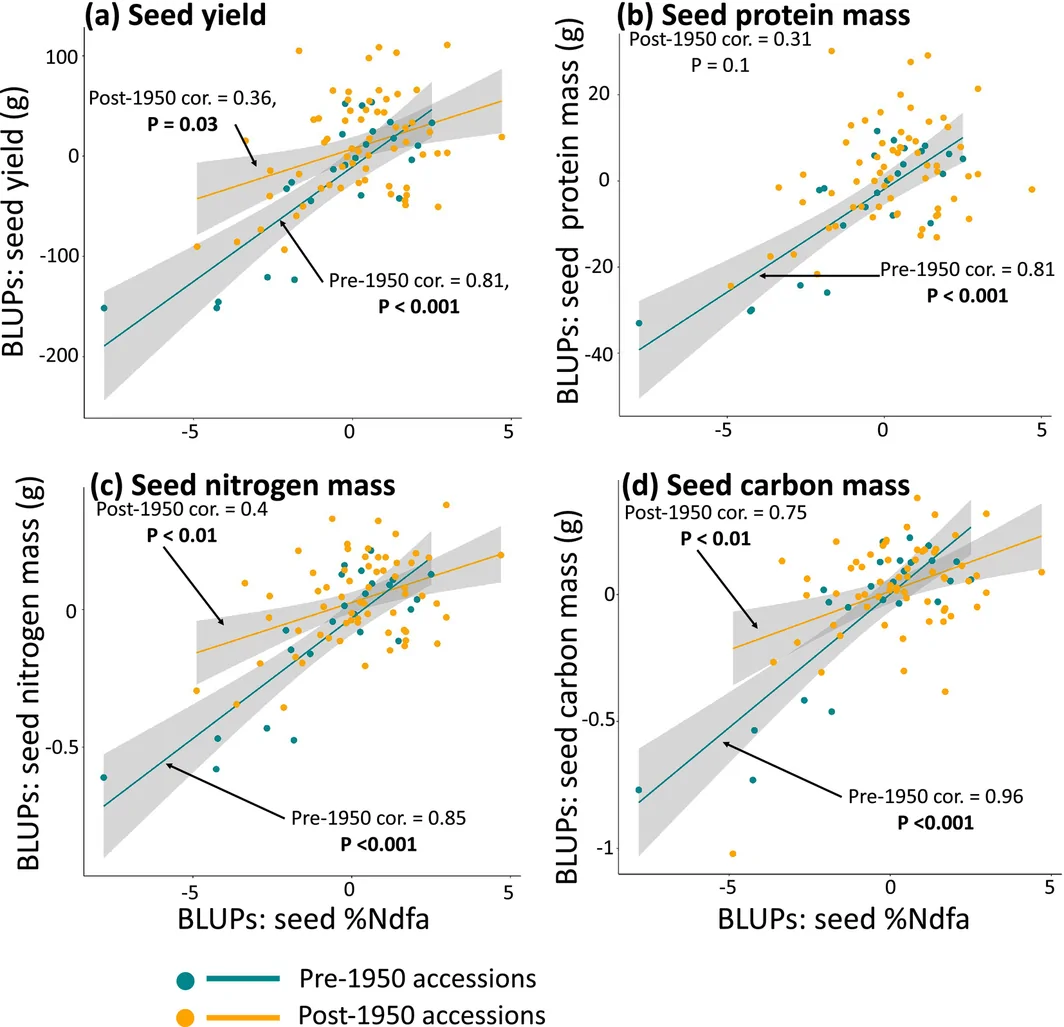
Positive genetic correlations were found between seed %Ndfa and (a) seed yield, (b) total seed protein mass, (c) total seed nitrogen mass and (d) total seed carbon mass, for pre‐1950 accessions (blue) and post‐1950 accessions (orange).

**FIGURE 3:**
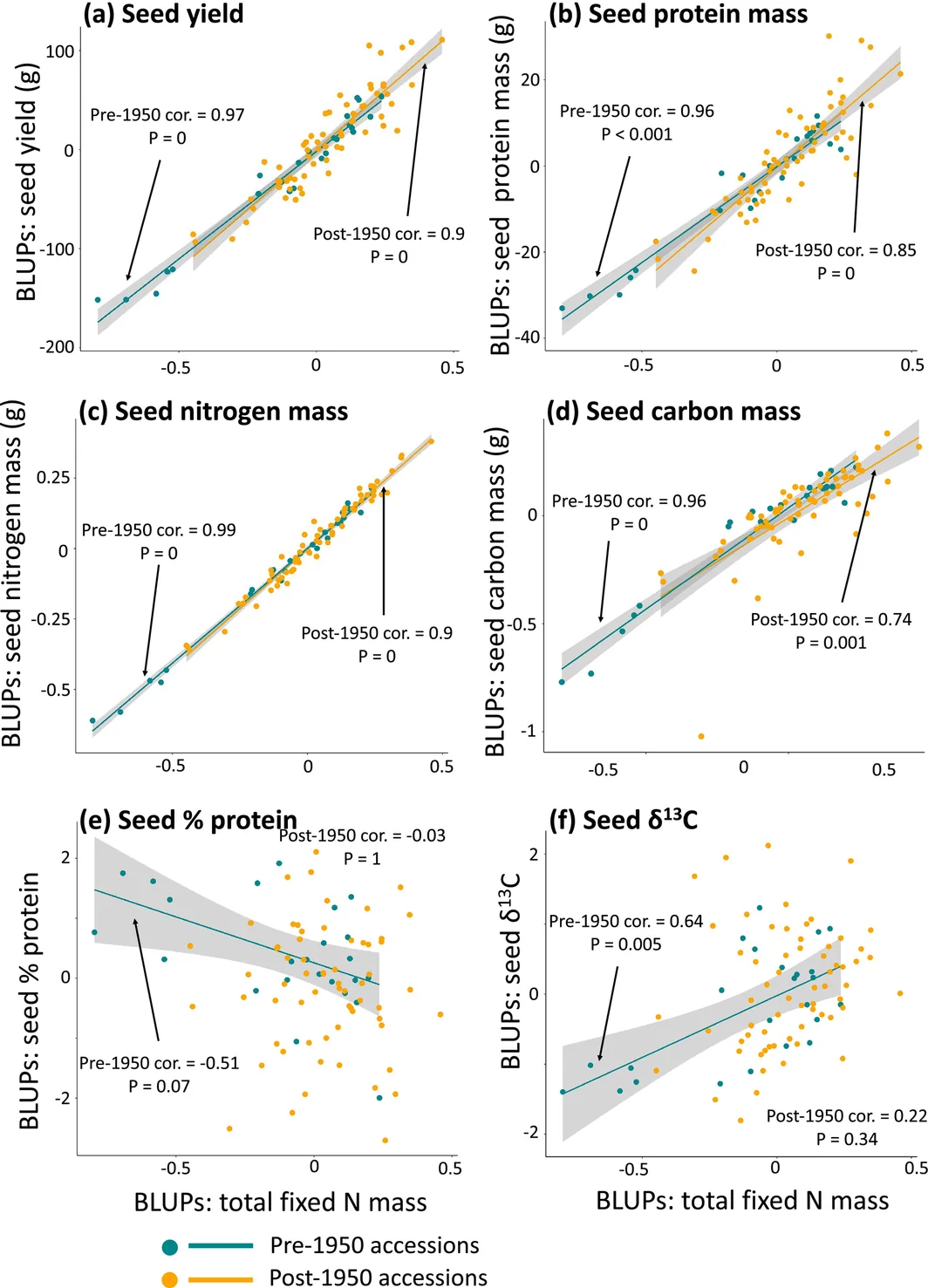
Positive genetic correlations were found in seed tissues between total fixed N mass and (a) seed yield, (b) total protein mass, (c) total nitrogen mass and (d) total carbon mass. There was a marginally significant negative genetic correlation between total fixed N mass and seed % protein (e) for pre‐1950 accessions, but not post‐1950 accessions. A significant positive correlation was found between total fixed N mass and seed δ13C (f) for pre‐1950 accessions, but not post‐1950 accessions.

For individual plant traits collected in 2020 prior to storm damage, there was a positive genetic correlation between leaf %Ndfa and shoot weight (*r*(85) = 0.64, *p* < 0.001, Table S5, Figure 4a) and between leaf %Ndfa and shoot N weight (*r*(85) = 0.65, *p* < 0.001, Table S5, Figure 4b) for domesticated pea. Leaf %Ndfa did not correlate with shoot % by weight N, root weight or nodule count for domesticated pea. Among the wild accessions grown in 2020, we did not detect a genetic correlation between leaf %Ndfa and shoot weight (Table S9, Figure 3a), nor between leaf %Ndfa and other phenotypes measured on individual plants.

**FIGURE 4:**
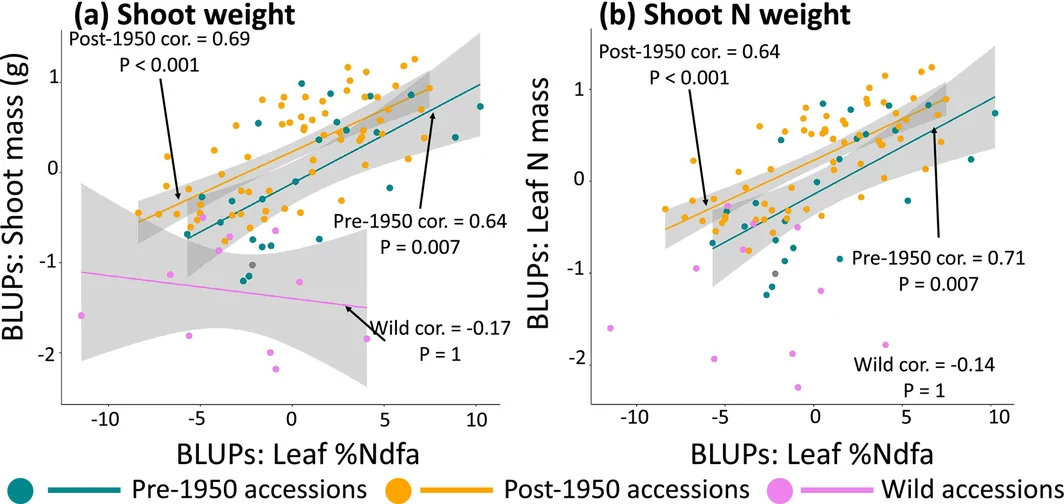
Positive genetic correlations were found between leaf %Ndfa and shoot mass (a) and leaf %Ndfa and leaf N mass (b) for pre‐1950 (blue) and post‐1950 (orange) accessions, while leaf %Ndfa and shoot mass were negatively genetically correlated in the wild accessions (violet).

### 3.2 Genetic Correlations in Pre‐ and Post‐ 1950 Age Groups

We also evaluated genetic correlations in groups of accessions registered with the USDA either before or after 1950. For the plot‐aggregated data, in both pre‐ and post‐1950 age groups there was a positive correlation between seed yield and seed %Ndfa, although the pre‐1950 correlation coefficient (pre‐150 *r*(22) = 0.81, *p* < 0.001, Table S6, Figure 2) was greater than the post‐1950 correlation coefficient (post‐1950 r(61) = 0.36, *p* = 0.03, Table S6, Figure 2). The difference between the pre‐ and post‐1950 correlations was significant (Fisher's *z* = 3.03, *p* = 0.002, Figure 2, Table S6). Similarly, there were stronger correlations in the pre‐1950 age group than the post‐1950 age group between seed %Ndfa and seed protein weight (Fisher's *z* = 3.26, *p* = 0.001, Figure 3, Table S6), N weight (Fisher's *z* = 3.28, *p* = 0.001, Figure 2, Table S6) and C weight (Fisher's *z* = 3.39, *p* < 0.001, Figure 2, Table S6). However, with the lowest‐yielding pre‐1950 accessions removed, there was no difference between the yield‐%Ndfa correlations for pre‐ and post‐1950 age groups (Table S7). Further, the GLMM testing the effect of the yield BLUPs: age group interaction on the seed %Ndfa BLUPs found no difference in the slopes of the pre‐ and post‐1950 yield‐%Ndfa correlations (Table S10). The pre‐ and post‐1950 age groups showed no detectable differences in how their seed %Ndfa correlated with their seed δ^13^C, protein concentration, % N weight or % C weight. In the single‐plant measurements from 2020, there were no detectable differences in correlations between the pre‐ and post‐1950 age groups (Table S8). We did not contrast pre‐ and post‐1950 correlations using Model C bivariate models, since the pre‐1950 models returned correlation coefficients greater than 1, which we have chosen not to report. However, in all of the bivariate models that included pre‐ and post‐1950 accessions, age group had a significant effect (Table S4).

### 3.3 Effect of Age Group on Yield and Symbiosis Traits

To assess how yield and symbiosis traits have changed over time, we used Model B to test the effect of age group on each trait, while accounting for the effect of accession, year and block. There was a marginal increase in seed %Ndfa in the post‐1950 accessions (*χ* ^2^: 3.23, *p* = 0.07, Figure 5a, Table S3). Weight‐based traits had higher means in the post‐1950 group than in the pre‐1950 group, including seed yield (*χ* ^2^: 3.23, *p* = 0.009, Figure 5b, Table S3), total fixed N weight (*χ* ^2^: 6.48, *p* = 0.01, Figure 5c, Table S3), protein weight (*χ* ^2^: 5.05, *p* = 0.02, Figure 5d, Table S3), C weight (*χ* ^2^: 5.39, *p* = 0.02, Figure 5e, Table S3) and, marginally, N weight (*χ* ^2^: 3.35, *p* = 0.06, Figure 5f, Table S3). Seed δ^13^C was also greater in the post‐1950 group (*χ* ^2^: 8.05, *p* = 0.004, Figure 5, Table S3), which could indicate higher water use efficiency in the newer accessions. Despite having higher total seed protein weight, seed protein concentration was lower in the post‐1950 age group (*χ* ^2^: 5.41, *p* = 0.01, Figure 5h, Table S3).

**FIGURE 5:**
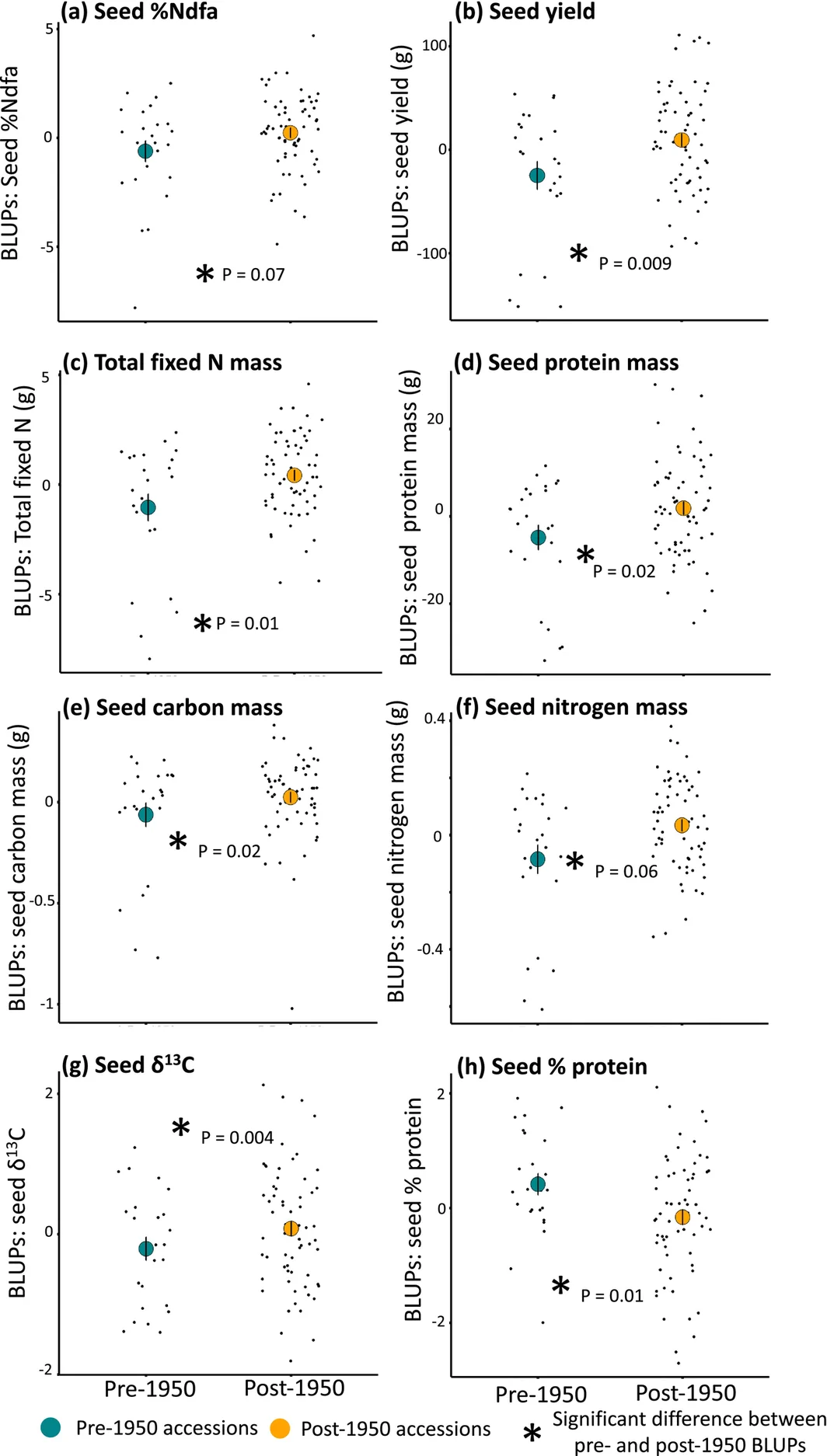
Seed yield (b), protein mass (d) carbon mass (e), nitrogen mass (f), and seed δ13C (g) were greater in post‐1950 accessions than pre‐1950 accessions. Seed %Ndfa (a) and total fixed N mass (c) were marginally greater in the post‐1950 accessions than in post‐1950 accessions. Seed % protein (h) was greater in pre‐1950 accessions than in post‐1950 accessions.

For the single‐plant measurements in 2020, we compared wild pea accessions to pre‐ and post‐1950 age groups of domesticated pea. Most phenotypes had greater means in the domesticated peas, including leaf %Ndfa (*χ* ^2^: 7.95, *p* = 0.04, Figure 6a, Table S9), shoot weight (*χ* ^2^: 53.7, *p* < 0.001, Figure 6b, Table S9), shoot fixed N weight (*χ* ^2^: 46.1, *p* < 0.001, Figure 6c, Table S9) and shoot N weight (*χ* ^2^: 51.8, *p* < 0.001, Figure 6d, Table S9) (seed protein concentration was not measured in wild accessions). Although shoot N weight was greater in domesticated accessions, leaf % N was greater in the wild peas (*χ* ^2^: 8.83, *p* = 0.03, Figure 6e, Table S9). Domesticated root weight was only greater than wild root weight for the post‐1950 age group (*χ* ^2^: 53.7, *p* = 0.01, Figure 6f, Table S9). Nodule count was indistinguishable among the groups of accessions.

**FIGURE 6:**
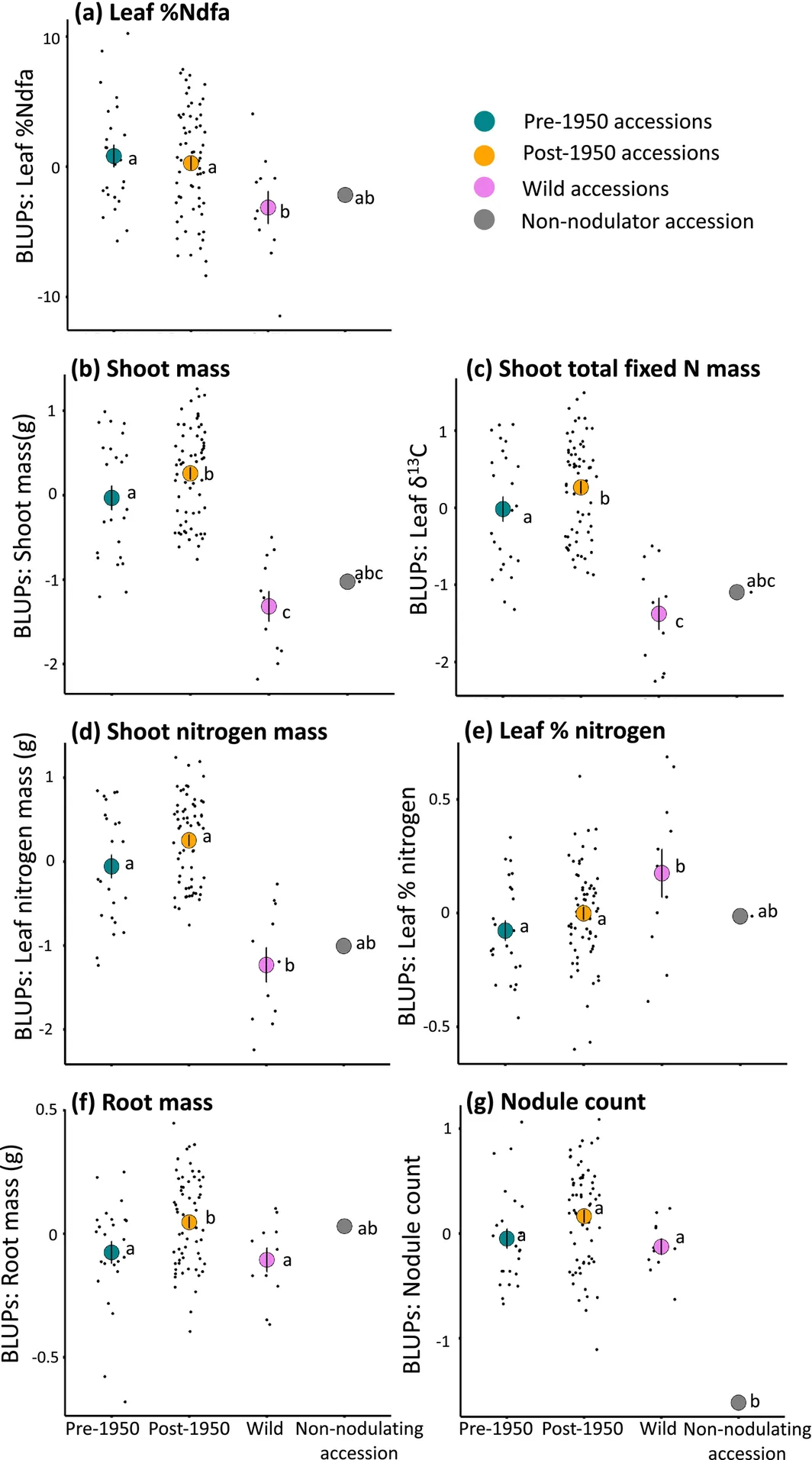
Domesticated accessions had higher mean values than wild accessions for leaf %Ndfa (a), shoot mass (b), shoot total fixed N mass (c), and shoot N mass (d). Wild accessions had greater leaf % N (e) than domesticated accessions. Post‐1950 accessions had greater root mass (f) than pre‐1950 accessions and wild accessions. Nodule count (g) did not differ significantly between accessions (other than the non‐nodulating accession).

Using Model B (excluding the experimental year effect), we tested the effect of pre‐ or post‐1950 age group on the single‐plant phenotypes from 2020. Age group effects on the single‐plant measurements were similar to those observed in plot‐aggregated measurements from 2019 and 2021: shoot weight (*χ* ^2^: 4.06, *p* = 0.04, Figure 6b, Table S5), shoot N weight (*χ* ^2^: 5.18, *p* < 0.001, Figure 6d, Table S5) and root weight (*χ* ^2^: 6.74, *p* = 0.009, Figure 6f, Table S5) were greater in the post‐1950 age group than the pre‐1950 age group.

## 4 Discussion

### 4.1 Yield and N_2_ Fixation Are Positively Genetically Correlated

We find that higher‐yielding pea accessions obtain a greater proportion of their N from symbiotic N_2_ fixation rhizobia than do lower‐yielding accessions. This positive genetic correlation between N_2_ fixation and yield in pea predicts that selection for higher yield will enhance or at least maintain the mutualistic symbiosis with rhizobia. Post‐1950 cultivars have higher mean yields, a greater mass of symbiotically fixed N_2_ and marginally greater %Ndfa means than pre‐1950 cultivars. Given these improvements in mean trait values and that the slope of the correlation between yield and %Ndfa does not differ between pre‐ and post‐1950 accessions, we infer that modern artificial selection for yield appears to have maintained or even increased peas' ability to fix N_2_ with rhizobia, rather than yield gains coming at a cost to legume investment in rhizobial symbiosis (West et al. 2002; Porter and Sachs 2020). Therefore, it appears to be feasible for artificial selection for both yield and symbiotic N_2_ fixation in pea. However, even high‐yielding pea accessions display abundant standing genetic variation in symbiotic N_2_ fixation. It will be critical for crop improvement efforts to evaluate both yield and symbiosis function to avoid inadvertently selecting high‐yielding lineages with low symbiotic N_2_ fixation in the future.

### 4.2 Genetic Correlations in Pre‐ and Post‐1950 Pea Accessions

Both pre‐ and post‐1950 accessions showed positive genetic correlations between yield and symbiosis. The 20th century was a period of intense crop improvement (Bradshaw 2017) with large increases in yields after 1950, coinciding with the ‘Green Revolution’ and modern agricultural practices (Brancourt‐Hulmel et al. 2003; Jin et al. 2010). The 1950s saw the proliferation of artificial fertiliser use and the formation of organisations like the National Pea Improvement Association, so we sought to examine the results of breeding before and after these developments. Positive yield‐ N_2_ fixation correlations appear weaker among post‐1950 cultivars, which could be interpreted as a form of symbiotic disruption, similar to that observed for mycorrhizal symbioses over the course of wheat improvement (Hetrick et al. 1993; Zhu et al. 2001). However, we did not detect differences in the slope of correlations for pre‐ and post‐1950 accessions, so while N_2_ fixation rates may not be as strongly predictive of yield in post‐1950 peas, the underlying positive genetic relationship between yield and N_2_ fixation is indistinguishable between older and newer accessions. We note that low‐yielding accessions are influential to these findings: when the five lowest‐yielding pre‐1950 accessions are excluded from the comparison, the yield‐symbiosis correlations do not differ between the pre‐ and post‐1950 age groups. Further work across a greater number of accessions is thus needed for more robust inference on evolutionary dynamics for these critical genetic correlations over the course of crop improvement.

### 4.3 Correlations Between Symbiotic N_2_ Fixation and Protein

Higher protein yield and concentration are primary breeding goals in pea (Coyne et al. 2005) to facilitate the use of plant‐based protein in food production (Burger and Zhang 2019; Lu et al. 2020). However, breeding gains in protein content often come at a cost to other traits, such as trade‐offs between yield and % protein content in pea and faba bean (Tar'an et al. 2004; Krajewski et al. 2012; Peltonen‐Sainio et al. 2011; Curti et al. 2018; Skovbjerg et al. 2020) and between oil and protein in rapeseed (Peltonen‐Sainio et al. 2011). Overall, we did not detect a genetic correlation between the proportion of seed N derived from N_2_ fixation (%Ndfa) and the proportion of protein in seeds by mass, though pre‐1950 accessions show a marginal negative genetic correlation between the two. This lack of strong trade‐offs, especially in newer accessions, is promising for pea germplasm improvement. Furthermore, the positive genetic correlations between %Ndfa and total seed N and protein mass indicates that section for increased pea protein production could even increase N_2_ fixation, especially under symbiotically informed breeding practices (Porter and Sachs 2020; Rog et al. 2026).

### 4.4 Superior Symbiotic Outcomes in Crop Peas Compared to Wild Peas

Theoretical (Porter and Sachs 2020) and empirical (Martín‐Robles et al. 2018) studies posit that wild plants may benefit more from soil mutualists than do their domesticated relatives due to relaxed selection in fertile agricultural conditions or trade‐offs with traits under artificial selection. In contrast to this prediction, we observed higher N_2_ fixation rates as %Ndfa in domesticated compared to wild accessions. Furthermore, wild and pre‐ and post‐1950 domesticated peas have indistinguishable numbers of nodules on 6‐week‐old root systems, even though domesticated pea has substantially greater above‐ground biomass than wild pea. Thus, domesticated pea accessions obtain a greater N_2_ fixation from the same number of nodules as wild peas, consistent with a scenario in which domestication has increased the efficiency of the mutualism in peas (Muñoz et al. 2016). We did not detect a genetic correlation between %Ndfa and shoot weight among wild peas, but our small sample limits inference about the effect of domestication on yield‐symbiosis correlations. Lower symbiotic function in wild peas could reflect low compatibility with the rhizobia or other conditions in agricultural soil and test of symbiotic function with rhizobia from their native range and in native habitats could be informative (Terpolilli et al. 2008; Drew and Ballard 2010; Kazmierczak et al. 2017; Rigg et al. 2021; Carlson et al. 2023).

### 4.5 Weather Extremes Can Disrupt the Yield‐Symbiosis Correlation

In 2020, an extreme storm disrupted the genetic correlation between yield and symbiosis apparent in other years, highlighting the sensitivity of symbiosis trait genetic correlations to disruption by extreme weather events. Increasing weather extremes are pervasive threats to agriculture (Aydinalp et al. 2008; Lesk et al. 2016; Vogel et al. 2019) and present a particular concern for plant breeding, since climatic variation can reduce the efficacy of selection and genetic gains made by breeding programs (Xiong et al. 2022). Years such as 2020, where we detected negligible genetic variation in N_2_ fixation and no correlation with yield, could interfere with efforts to improve symbiotic benefits in peas. Damage from May 2020's rainstorm was directly predictive of yields: the most storm‐damaged plots had the lowest yield. However, the most storm‐damaged plots also had the highest N_2_ fixation. This could arise if damaged plants invested more in symbiosis, as increases in nodule biomass in response to abiotic stress can occur (Wurzburger and Ford Miniat 2014). Alternatively, the positive correlation between leaf N_2_ fixation and shoot weight prior to the storm indicates that pea plants with higher N_2_‐fixation rates were larger and therefore may have been more susceptible to damage from heavy rain.

### 4.6 Future Directions

Our findings point to the need for additional experiments to discern the generality of our findings. The relationship between yield and symbiotic traits may depend on the makeup and the density of the rhizobial community and the soil chemistry at a site (Terpolilli et al. 2008; Drew and Ballard 2010; Kazmierczak et al. 2017; Rigg et al. 2021). In addition, soil fertility can impact the benefits of symbiosis. The MP3 peas were N fertilised, as is recommended to maximise pea yields (Pavek & NRCS Pullman Plant Materials Center, 2012), which can reduce the benefits of the rhizobial mutualism, as it lowers a plant's demand for fixed N (Regus et al. 2016). Our experiment was fertilised with an average of 39.2 kg ha^−1^ N each year, which is lower than the recommended 50 kg ha^−1^ (Pavek & NRCS Pullman Plant Materials Center 2012), but could prevent peas from gaining maximal benefit from rhizobia. More broadly distributed studies, potentially with rhizobial inoculation (Vessey 2004; Ahmed et al. 2007), are required to determine whether the patterns we observe are consistent across locations.

## 5 Conclusion

Our findings provide a novel *genetic* perspective on yield and symbiosis trait correlations of more direct relevance to crop improvement than prior observations of positive *phenotypic* correlations between yield and symbiosis traits (Dhillon et al. 2022). This is because heritable, genetically based trait correlations can inform multi‐trait breeding efforts. The positive genetic correlation between yield and the proportion of N that plants get from rhizobia in both pre‐ and post‐1950 accessions suggests that selection for yield enhances or maintains the pea‐rhizobium mutualism, rather than disrupting it. Furthermore, abundant genetic variation in N_2_ fixation indicates that there is great potential for simultaneous improvement of both pea symbiotic N_2_ fixation and pea yields. In 1 year of our study, a major storm damaged plants and eliminated detectable genetic variation and correlations that were apparent under normal conditions. As extreme weather events become more common, disruption of genetic correlations between yield and symbiosis could hamper breeding efforts due to stochastic damage and/or reduction of the benefits of symbiosis for crops. Nevertheless, our findings suggest that symbiotically informed breeding in crops is a promising tool to simultaneously meet demands for increased yield, seed protein concentration and nitrogen fixation and thus reduce agricultural dependence on environmentally detrimental inorganic N fertiliser.

## Funding

Funding was provided by National Science Foundation DEB‐1943239, USDA‐AFRI‐A1102‐2026‐67,013‐45,699 and the WSDA‐Specialty Crop Block Grant Program (to Stephanie Porter) and the United States Department of Agriculture Agricultural Research Service Pulse Crop Health Initiative (to Clarice Coyne).

## Conflicts of Interest

The authors declare no conflicts of interest.

## Supporting information


**Table S1:** List of accessions used in experiment. Year received by NPGS refers to the year that the accession was catalogued by the National Plant Germplasm System.
**Table S2:** Fertiliser applications by year.
**Figure S1:** May 2020 was an extreme outlier in rainfall due to the rainstorm on the 20th. Storm damage severity correlated negatively with seed yield. Daily rainfall totals per growing season month for 2019, 2020 and 2021 (a) and the correlation between seed yield and storm damage severity (b).
**Table S3:** Correlations between seed %Ndfa and yield traits and the effect of age group on traits, with data from 2019 and 2021. Pearson coefficients and *p* values for each %Ndfa‐yield trait correlation are shown, as well as the *χ* ^2^ value and *p* value of likelihood ratio tests of the effect of age group on each trait.
**Table S4:** Correlations between seed %Ndfa and yield traits and the effect of age group on the correlations, generated from bivariate models using data from 2019 and 2021. Bivariate models included the listed phenotype and seed %Ndfa as response variables. Correlation coefficients come from the variance–covariance matrix for the accession random effect. *p*‐values for the correlations come from likelihood ratio tests, where the null model included a constraint matrix that limited the accession random effect to 0 covariance. *p*‐values for the effect of age group come from likelihood ratio tests, where the null model does not include the age group effect.
**Table S5:** Correlations between seed %Ndfa and yield traits and the effect of age group on traits, with data from 2020. Pearson coefficients and *p* values for each %Ndfa‐yield trait correlation are shown, as well as the *χ* ^2^ value and *p* value of likelihood ratio tests of the effect of age group on each trait.
**Table S6:** Contrasts between pre‐ and post‐1950 seed %Ndfa‐ yield trait correlations, with data from 2019 and 2021. Pearson coefficients and *p* values of each pre‐ and post‐1950 correlation are shown. The *p* values of the contrasts between each pre‐ and post‐1950 correlation are also shown.
**Table S7:** Contrasts between pre‐ and post‐1950 seed %Ndfa‐ yield trait correlations, with the five lowest‐yielding pre‐1950 accessions excluded, with data from 2019 and 2021. Pearson coefficients and *p* values of each pre‐ and post‐1950 correlation are shown. The *p* values of the contrasts between each pre‐ and post‐1950 correlation are also shown.
**Table S8:** Contrasts between pre‐ and post‐1950 seed %Ndfa‐ yield trait correlations, with data from 2020. Pearson coefficients and *p* values of each pre‐ and post‐1950 correlation are shown. The *p* values of the contrasts between each pre‐ and post‐1950 correlation are also shown.
**Table S9:** Correlations between leaf %Ndfa and yield traits for wild accessions in 2020 and their contrasts with the domesticated accessions. Pearson coefficients and *p* values for each %Ndfa‐yield trait correlation are shown, as well as the *χ* ^2^ value and *p* value of likelihood ratio tests of the effect of domestication status on each trait.
**Table S10:** GLMM comparing the slope of the yield‐N fixation genetic correlation between pre‐ and post‐1950 accessions. The significant effect of the yield BLUPs shows the positive genetic correlation between yield and N fixation. The insignificant effect of the yield BLUPs:Age group interaction shows that the slope of the yield‐N fixation genetic correlation does not differ between the pre‐ and post‐1950 age groups.

## Data Availability

Data and code for this study are archived on Harvard Dataverse.

## References

[eva70330-bib-0001] Ahmed, R. , A. R. Solaiman , N. K. Halder , M. A. Siddiky , and M. S. Islam . 2007. “Effect of Inoculation Methods of Rhizobium on Yield Attributes Yield and Protein Content in Seed of Pea.” Journal of Soil and Nature 1: 30–35.

[eva70330-bib-0002] Alves, B. J. R. , R. M. Boddey , and S. Urquiaga . 2003. “The Success of BNF in Soybean in Brazil.” In Plant and Soil, 1–9. Springer.

[eva70330-bib-0003] Aydinalp, C. , M. S. Cresser , and M. S. Cresser . 2008. “The Effects of Global Climate Change on Agriculture Agriculture Land Use Change (Including Biomass Burning) the Effects of Global Climate Change on Agriculture.” Journal of Agricultural & Environmental Sciences 3: 672–676.

[eva70330-bib-0005] Bennett, A. E. , T. J. Daniell , and P. J. White . 2013. “Benefits of Breeding Crops for Yield Response to Soil Organisms.” In Molecular Microbial Ecology of the Rhizosphere: Volume 1, 17–27. John Wiley & Sons, Inc.

[eva70330-bib-0006] Bradshaw, J. E. 2017. “Plant Breeding: Past, Present and Future.” Euphytica 213: 1–12.

[eva70330-bib-0007] Brancourt‐Hulmel, M. , G. Doussinault , C. Lecomte , P. Bérard , B. Le Buanec , and M. Trottet . 2003. “Genetic Improvement of Agronomic Traits of Winter Wheat Cultivars Released in France From 1946 to 1992.” Crop Science 43: 37–45.

[eva70330-bib-0008] Brooks, M. E. J. K. K. , K. van Benthem , A. Magnusson , et al. 2017. “glmmTMB Balances Speed and Flexibility Among Packages for Zero‐Inflated Generalized Linear Mixed Modeling.” R Journal 9: 378–400.

[eva70330-bib-0009] Burger, T. G. , and Y. Zhang . 2019. “Recent Progress in the Utilization of Pea Protein as an Emulsifier for Food Applications.” Trends in Food Science & Technology 86: 25–33.

[eva70330-bib-0010] Carlson, C. , E. Akçay , and B. Morsky . 2023. “The Evolution of Partner Specificity in Mutualisms.” Evolution 77: 881–892. 10.1093/evolut/qpac056 36745019

[eva70330-bib-0011] Chen, Y. H. , R. Gols , and B. Benrey . 2015. “Crop Domestication and Its Impact on Naturally Selected Trophic Interactions.” Annual Review of Entomology 60: 35–58. 10.1146/annurev-ento-010814-020601 25341108

[eva70330-bib-0012] Cheng, P. , W. Holdsworth , Y. Ma , et al. 2015. “Association Mapping of Agronomic and Quality Traits in USDA Pea Single‐Plant Collection.” Molecular Breeding 35: 1–13.

[eva70330-bib-0013] Collet, J. M. , S. Nidelet , and S. Fellous . 2023. “Genetic Independence Between Traits Separated by Metamorphosis Is Widespread but Varies With Biological Function.” Proceedings of the Royal Society B: Biological Sciences 290. 10.1098/rspb.2023.1784. PMC10645066 37935368

[eva70330-bib-0014] Córdova‐Campos, O. , R. M. Adame‐Álvarez , J. A. Acosta‐Gallegos , and M. Heil . 2012. “Domestication Affected the Basal and Induced Disease Resistance in Common Bean ( *Phaseolus vulgaris* ).” European Journal of Plant Pathology 134: 367–379.

[eva70330-bib-0015] Covarrubias‐Pazaran, G. 2016. “Genome‐Assisted Prediction of Quantitative Traits Using the R Package Sommer.” PLoS One 11: e0156744. 10.1371/journal.pone.0156744 PMC4894563 27271781

[eva70330-bib-0016] Cox, A. , L. Boots‐Haupt , K. Brasier , R. Riar , and H. Zakeri . 2022. “Using δ15N to Screen for Nitrogen Fixation: Reference Plant Position and Species.” Agronomy Journal 114: 1842–1850.

[eva70330-bib-0017] Coyne, C. J. , M. A. Grusak , L. Razai , and B.‐K. Baik . 2005. “Variation for Pea Seed Protein Concentration in the USDA Pisum Core Collection.” Pisum Genetics 37: 5–9.

[eva70330-bib-0019] Curti, R. N. , M. C. Sanahuja , S. M. Vidueiros , A. N. Pallaro , and H. D. Bertero . 2018. “Trade‐Off Between Seed Yield Components and Seed Composition Traits in Sea Level Quinoa in Response to Sowing Dates.” Cereal Chemistry 95: 734–741.

[eva70330-bib-0021] Dhillon, L. K. , D. Lindsay , T. Yang , et al. 2022. “Biological Nitrogen Fixation Potential of Pea Lines Derived From Crosses With Nodulation Mutants.” Field Crops Research 289: 108731.

[eva70330-bib-0022] Diedenhofen, B. , and J. Musch . 2015. “Cocor: A Comprehensive Solution for the Statistical Comparison of Correlations.” PLoS One 10: e0121945. 10.1371/journal.pone.0121945 PMC4383486 25835001

[eva70330-bib-0023] Drew, E. A. , and R. A. Ballard . 2010. “Improving N_2_ Fixation From the Plant Down: Compatibility of *Trifolium subterraneum* L. Cultivars With Soil Rhizobia Can Influence Symbiotic Performance.” Plant and Soil 327: 261–277.

[eva70330-bib-0024] Dwivedi, S. L. , K. L. Sahrawat , H. D. Upadhyaya , et al. 2015. “Advances in Host Plant and Rhizobium Genomics to Enhance Symbiotic Nitrogen Fixation in Grain Legumes.” Advances in Agronomy 129: 1–116.

[eva70330-bib-0025] Evans, L. T. , J. Bingham , R. D. Blackwell , M. A. Ford , C. L. Morgan , and M. Taylor . 1980. “Genetic Improvements in Winter Wheat Yields Since 1900 and Associated Physiological Changes.” Journal of Agricultural Science 94: 675–689.

[eva70330-bib-0027] Hadfield, J. D. , A. J. Wilson , D. Garant , B. C. Sheldon , and L. E. B. Kruuk . 2010. “The Misuse of BLUP in Ecology and Evolution.” American Naturalist 175: 116–125. 10.1086/648604 19922262

[eva70330-bib-0028] Hartig, F. 2022. “DHARMa: Residual Diagnostics for Hierarchical (Multi‐Level/Mixed) Regression Models.”

[eva70330-bib-0029] Herridge, D. F. , J. F. Holland , I. A. Rose , and R. J. Redden . 1998. “Selection and Breeding of Grain Legumes in Australia for Enhanced Nodulation and N{sub 2} Fixation.”

[eva70330-bib-0030] Herridge, D. F. , M. J. Robertson , B. Cocks , et al. 2005. “Low Nodulation and Nitrogen Fixation of Mungbean Reduce Biomass and Grain Yields.” Australian Journal of Experimental Agriculture 45: 269–277.

[eva70330-bib-0031] Hetrick, B. A. D. , G. W. T. Wilson , and T. S. Cox . 1993. “Mycorrhizal Dependence of Modern Wheat Cultivars and Ancestors: A Synthesis.” Canadian Journal of Botany 71: 512–518.

[eva70330-bib-0032] Hill, W. G. , and T. F. C. Mackay . 2004. “D. S. Falconer and Introduction to Quantitative Genetics.” Genetics 167: 1529–1536. 10.1093/genetics/167.4.1529 PMC1471025 15342495

[eva70330-bib-0033] Holdsworth, W. L. , E. Gazave , P. Cheng , et al. 2017. “A Community Resource for Exploring and Utilizing Genetic Diversity in the USDA Pea Single Plant Plus Collection.” Horticulture Research 4: 17017. 10.1038/hortres.2017.17 PMC5405346 28503311

[eva70330-bib-0035] Jacott, C. N. , J. D. Murray , and C. J. Ridout . 2017. “Trade‐Offs in Arbuscular Mycorrhizal Symbiosis: Disease Resistance, Growth Responses and Perspectives for Crop Breeding.” Agronomy 7: 75.

[eva70330-bib-0036] Jermyn, W. A. , and A. E. Slinkard . 1977. “Variability of Percent Protein and Its Relationship to Seed Yield and Seed Shape in Peas.” Legume Research 1: 33–37.

[eva70330-bib-0037] Jin, J. , X. Liu , G. Wang , et al. 2010. “Agronomic and Physiological Contributions to the Yield Improvement of Soybean Cultivars Released From 1950 to 2006 in Northeast China.” Field Crops Research 115: 116–123.

[eva70330-bib-0038] Kaler, A. S. , S. K. Bazzer , A. Sanz‐Saez , J. D. Ray , F. B. Fritschi , and L. C. Purcell . 2018. “Carbon Isotope Ratio Fractionation Among Plant Tissues of Soybean.” Plant Phenome Journal 1: 1–6.

[eva70330-bib-0039] Kazmierczak, T. , M. Nagymihaly , F. Lamouche , et al. 2017. “Specific Host‐Responsive Associations Between *Medicago truncatula* Accessions and *Sinorhizobium* Strains.” Molecular Plant‐Microbe Interactions 30: 399–409. 10.1094/MPMI-01-17-0009-R 28437159

[eva70330-bib-0040] Khazaei, H. , G. Hawkins , and A. Vandenberg . 2021. “Historical Review of Faba Bean Improvement in Western Canada.” Legume Science 3: e92. 10.1002/leg3.75 PMC8700193 34977588

[eva70330-bib-0043] Kosterin, O. E. , O. O. Zaytseva , V. S. Bogdanova , and M. J. Ambrose . 2010. “New Data on Three Molecular Markers From Different Cellular Genomes in Mediterranean Accessions Reveal New Insights Into Phylogeography of *Pisum sativum* L. Subsp. Elatius (Bieb.) Schmalh.” Genetic Resources and Crop Evolution 57: 733–739.

[eva70330-bib-0045] Krajewski, P. , J. Bocianowski , M. Gawłowska , et al. 2012. “QTL for Yield Components and Protein Content: A Multienvironment Study of Two Pea ( *Pisum sativum* L.) Populations.” Euphytica 183: 323–336.

[eva70330-bib-0049] Lesk, C. , P. Rowhani , and N. Ramankutty . 2016. “Influence of Extreme Weather Disasters on Global Crop Production.” Nature 529: 84–87. 10.1038/nature16467 26738594

[eva70330-bib-0050] Liu, L. , J. D. Knight , R. L. Lemke , and R. E. Farrell . 2019. “A Side‐By‐Side Comparison of Biological Nitrogen Fixation and Yield of Four Legume Crops.” Plant and Soil 442: 169–182.

[eva70330-bib-0051] Lu, Z. X. , J. F. He , Y. C. Zhang , and D. J. Bing . 2020. “Composition, Physicochemical Properties of Pea Protein and Its Application in Functional Foods.” Critical Reviews in Food Science and Nutrition 60: 2593–2605. 10.1080/10408398.2019.1651248 31429319

[eva70330-bib-0052] Martín‐Robles, N. , A. Lehmann , E. Seco , R. Aroca , M. C. Rillig , and R. Milla . 2018. “Impacts of Domestication on the Arbuscular Mycorrhizal Symbiosis of 27 Crop Species.” New Phytologist 218: 322–334. 10.1111/nph.14962 29281758

[eva70330-bib-0054] Millar, N. , J. Piovia‐Scott , and S. S. Porter . 2023. “Impacts of Domestication on the Rhizobial Mutualism of Five Legumes Across a Gradient of Nitrogen‐Fertilisation.” Plant and Soil 491: 1–21.

[eva70330-bib-0055] Moyers, B. T. , P. L. Morrell , and J. K. McKay . 2018. “Genetic Costs of Domestication and Improvement.” Journal of Heredity 109: 103–116. 10.1093/jhered/esx069 28992310

[eva70330-bib-0056] Muñoz, N. , X. Qi , M.‐W. Li , et al. 2016. “Improvement in Nitrogen Fixation Capacity Could Be Part of the Domestication Process in Soybean.” Heredity 117: 84–93. 10.1038/hdy.2016.27 PMC4949726 27118154

[eva70330-bib-0057] Nicolás, M. F. , C. A. Arrabal Arias , and M. Hungria . 2002. “Genetics of Nodulation and Nitrogen Fixation in Brazilian Soybean Cultivars.” Biology and Fertility of Soils 36: 109–117.

[eva70330-bib-0058] Nishida, H. , and T. Suzaki . 2018. “Nitrate‐Mediated Control of Root Nodule Symbiosis.” Current Opinion in Plant Biology 44: 129–136. 10.1016/j.pbi.2018.04.006 29684704

[eva70330-bib-0060] Pavek, P. , and NRCS Pullman Plant Materials Center U . 2012. “Plant Guide PEA *Pisum sativum* L. Plant Symbol = PISA6.”

[eva70330-bib-0061] Peltonen‐Sainio, P. , L. Jauhiainen , M. Hyövelä , and E. Nissilä . 2011. “Trade‐Off Between Oil and Protein in Rapeseed at High Latitudes: Means to Consolidate Protein Crop Status?” Field Crops Research 121: 248–255.

[eva70330-bib-0062] Peng, S. , R. C. Laza , R. M. Visperas , A. L. Sanico , K. G. Cassman , and G. S. Khush . 2000. “Grain Yield of Rice Cultivars and Lines Developed in The Philippines Since 1966.” Crop Science 40: 307–314.

[eva70330-bib-0063] Porter, S. S. , and J. L. Sachs . 2020. “Agriculture and the Disruption of Plant–Microbial Symbiosis.” Trends in Ecology & Evolution 35: 426–439. 10.1016/j.tree.2020.01.006 32294424

[eva70330-bib-0064] R Core Team . 2014. R Core Team (2014). R: A Language and Environment for Statistical Computing. R Foundation for Statistical Computing. URL. http://www.R‐project.org/.

[eva70330-bib-0065] Regus, J. U. , C. E. Wendlandt , R. M. Bantay , et al. 2016. “Nitrogen Deposition Decreases the Benefits of Symbiosis in a Native Legume.” Plant and Soil 414: 159–170.

[eva70330-bib-0066] Rigg, J. L. , A. T. Webster , D. M. Harvey , et al. 2021. “Cross‐Host Compatibility of Commercial Rhizobial Strains for New and Existing Pasture Legume Cultivars in South‐Eastern Australia.” Crop and Pasture Science 72: 652–665.

[eva70330-bib-0068] Rog, I. , S. Lutz , L. Fesenfeld , J. Hiltbrunner , H. Bouwmeester , and M. G. A. van der Heijden . 2026. “Breeding for Beneficial Microbial Associations.” Nature Communications 17: 7695. 10.1038/s41467-026-76260-6 PMC13434687 42547509

[eva70330-bib-0071] Sagan, M. , A. Messager , and G. Duc . 1993. “Specificity of the Rhizobium—Legume Symbiosis Obtained After Mutagenesis in Pea ( *Pisum sativum* L.).” New Phytologist 125: 757–761. 10.1111/j.1469-8137.1993.tb03925.x 33874456

[eva70330-bib-0072] Sari, H. , C. J. Coyne , M. A. Grusak , et al. 2025. “Genome‐Wide Association Study of Seed Mineral Nutrients in Peas ( *Pisum sativum* L.).” Plant Genome 18: e70137. 10.1002/tpg2.70137 PMC12516006 41077665

[eva70330-bib-0096] Shaw, R. G. 1986. “Response To Density In a Wild Population Of The Perennial Herb Salvia Lyrata: Variation Among Families.” Evolution 40, no. 3: 492–505. 10.1111/J.1558-5646.1986.TB00502.X. 28556324

[eva70330-bib-0074] Sikinarum, J. , P. Jaisil , S. Jogloy , B. Toomsan , T. Kesmala , and A. Patanothai . 2007. “Heritability and Correlation for Nitrogen (N2) Fixation and Related Traits in Peanut ( *Arachis hypogaea* L.).” Pakistan Journal of Biological Sciences 10: 1956–1962. 10.3923/pjbs.2007.1956.1962 19093432

[eva70330-bib-0076] Skovbjerg, C. K. , J. N. Knudsen , W. Füchtbauer , et al. 2020. “Evaluation of Yield, Yield Stability, and Yield–Protein Relationship in 17 Commercial Faba Bean Cultivars.” Legume Science 2: e39.

[eva70330-bib-0077] Smýkal, P. , C. J. Coyne , M. J. Ambrose , et al. 2015. “Legume Crops Phylogeny and Genetic Diversity for Science and Breeding.” Critical Reviews in Plant Sciences 34: 43–104.

[eva70330-bib-0078] Tamagno, S. , V. O. Sadras , J. W. Haegele , P. R. Armstrong , and I. A. Ciampitti . 2018. “Interplay Between Nitrogen Fertilizer and Biological Nitrogen Fixation in Soybean: Implications on Seed Yield and Biomass Allocation.” Scientific Reports 8: 1–11. 10.1038/s41598-018-35672-1 PMC6269449 30504907

[eva70330-bib-0079] Tar'an, B. , T. Warkentin , D. J. Somers , et al. 2004. “Identification of Quantitative Trait Loci for Grain Yield, Seed Protein Concentration and Maturity in Field Pea ( *Pisum sativum* L.).” Euphytica 136: 297–306.

[eva70330-bib-0080] Terpolilli, J. J. , G. W. O'Hara , R. P. Tiwari , M. J. Dilworth , and J. G. Howieson . 2008. “The Model Legume *Medicago truncatula* A17 Is Poorly Matched for N2 Fixation With the Sequenced Microsymbiont *Sinorhizobium meliloti* 1021.” New Phytologist 179: 62–66. 10.1111/j.1469-8137.2008.02464.x 18422896

[eva70330-bib-0081] Unkovich, M. , D. Herridge , M. Peoples , et al. 2008. Measuring Plant‐Associated Nitrogen Fixation in Agricultural Systems, 132–188. Australian Center for International Agricultural Research.

[eva70330-bib-0082] Vessey, J. K. 2004. “Benefits of Inoculating Legume Crops With Rhizobia in the Northern Great Plains.” Crop Management 3: 1–8.

[eva70330-bib-0083] Vogel, E. , M. G. Donat , L. V. Alexander , et al. 2019. “The Effects of Climate Extremes on Global Agricultural Yields.” Environmental Research Letters 14: 054010.

[eva70330-bib-0086] West, S. A. , E. T. Kiers , E. L. Simms , and R. F. Denison . 2002. “Sanctions and Mutualism Stability: Why Do Rhizobia Fix Nitrogen?” Proceedings of the Royal Society of London. Series B: Biological Sciences 269: 685–694. 10.1098/rspb.2001.1878 PMC1690951 11934359

[eva70330-bib-0087] White, J. , J. Prell , E. K. James , and P. Poole . 2007. “Nutrient Sharing Between Symbionts.” Plant Physiology 144: 604–614. 10.1104/pp.107.097741 PMC1914197 17556524

[eva70330-bib-0089] Wurzburger, N. , and C. Ford Miniat . 2014. “Drought Enhances Symbiotic Dinitrogen Fixation and Competitive Ability of a Temperate Forest Tree.” Oecologia 174: 1117–1126. 10.1007/s00442-013-2851-0 24337710

[eva70330-bib-0091] Xiong, W. , M. Reynolds , and Y. Xu . 2022. “Climate Change Challenges Plant Breeding.” Current Opinion in Plant Biology 70: 102308. 10.1016/j.pbi.2022.102308 36279790

[eva70330-bib-0093] Zhu, Y. G. , S. E. Smith , A. R. Barritt , and F. A. Smith . 2001. “Phosphorus (P) Efficiencies and Mycorrhizal Responsiveness of Old and Modern Wheat Cultivars.” Plant and Soil 237: 249–255.

[eva70330-bib-0094] Zohary, D. 2004. “Unconscious Selection and the Evolution of Domesticated Plants.” Economic Botany 58: 5–10.

[eva70330-bib-0095] Zou, G. Y. 2007. “Toward Using Confidence Intervals to Compare Correlations.” Psychological Methods 12: 399–413. 10.1037/1082-989X.12.4.399 18179351

