# Supplementary material for: Positive Genetic Correlation Between Yield and Symbiotic Nitrogen Fixation in Pea
Source: Evol Appl. 2026 Oct 13;19(10):e70330. doi: 10.1111/eva.70330 (PMC13659271; doi:10.1111/eva.70330)
Supplement: Supplementary file 1 [file EVA-19-e70330-s001.docx]

**Supplement: Positive genetic correlation between yield and symbiotic nitrogen fixation in pea**

**Methods supplement**

**Table S1: List of accessions used in experiment.** Year received by NPGS refers to the year that the accession was catalogued by the National Plant Germplasm System.

**Table S2: Fertiliser applications by year**

**Results supplement**

**Fig. S1: May 2020 was an extreme outlier in rainfall due to the rainstorm on the 20^th^. Storm damage severity correlated negatively with seed yield.** Daily rainfall totals per growing season month for 2019, 2020, and 2021 (a), and the correlation between seed yield and storm damage severity (b).

**Table S3: Correlations between seed %Ndfa and yield traits, and the effect of age group on traits, with data from 2019 & 2021.** Pearson coefficients and P values for each %Ndfa-yield trait correlation are shown, as well as the χ^2^ value and P value of likelihood ratio tests of the effect of age group on each trait.

**Table S4: Correlations between seed %Ndfa and yield traits, and the effect of age group on the correlations, generated from bivariate models using data from 2019 & 2021.** Bivariate models included the listed phenotype and seed %Ndfa as response variables. Correlation coefficients come from the variance-covariance matrix for the accession random effect. P-values for the correlations come from likelihood ratio tests, where the null model included a constraint matrix that limited the accession random effect to 0 covariance. P-values for the effect of age group come from likelihood ratio tests, where the null model does not include the age group effect.

**Table S5: Correlations between seed %Ndfa and yield traits, and the effect of age group on traits, with data from 2020.** Pearson coefficients and P values for each %Ndfa-yield trait correlation are shown, as well as the χ^2^ value and P value of likelihood ratio tests of the effect of age group on each trait.

**Table S6: Contrasts between pre- and post-1950 seed %Ndfa- yield trait correlations, with data from 2019 & 2021.** Pearson coefficients and P values of each pre- and post-1950 correlation are shown. The P values of the contrasts between each pre- and post-1950 correlation are also shown.

**Table S7: Contrasts between pre- and post-1950 seed %Ndfa- yield trait correlations, with the five lowest-yielding pre-1950 accessions excluded, with data from 2019 & 2021.** Pearson coefficients and P values of each pre- and post-1950 correlation are shown. The P values of the contrasts between each pre- and post-1950 correlation are also shown.

**Table S8: Contrasts between pre- and post-1950 seed %Ndfa- yield trait correlations, with data from 2020.** Pearson coefficients and P values of each pre- and post-1950 correlation are shown. The P values of the contrasts between each pre- and post-1950 correlation are also shown.

**Table S9: Correlations between leaf %Ndfa and yield traits for wild accessions in 2020, and their contrasts with the domesticated accessions.** Pearson coefficients and P values for each %Ndfa-yield trait correlation are shown, as well as the χ^2^ value and P value of likelihood ratio tests of the effect of domestication status on each trait.

**Table S10: GLMM comparing the slope of the yield-N fixation genetic correlation between pre- and post-1950 accessions.** The significant effect of the yield BLUPs shows the positive genetic correlation between yield and N fixation. The insignificant effect of the yield BLUPs:Age group interaction shows that the slope of the yield-N fixation genetic correlation does not differ between the pre- and post-1950 age groups.
